# Borderline personality disorder traits and mentalising ability: The self-other social cognition paradox

**DOI:** 10.3389/fpsyt.2022.1023348

**Published:** 2022-10-20

**Authors:** Molly Kelly Grealy, Emmet Godfrey, Finn Brady, Erin Whyte O’Sullivan, Grace A. Carroll, Tom Burke

**Affiliations:** ^1^School of Psychology, National University of Ireland Galway, Galway, Ireland; ^2^School of Psychology, Queen’s University Belfast, Belfast, United Kingdom; ^3^Galway Neuroscience Centre, National University of Ireland Galway, Galway, Ireland

**Keywords:** borderline personality disorder traits, social cognition, mentalising, emotion recognition, empathy

## Abstract

**Objective:**

Borderline personality disorder (BPD) is a psychiatric condition characterised by a pervasive pattern of impulsivity, low self-image, and interpersonal conflicts. Previous findings indicate a mixed relationship between BPD and social cognition; little research as investigated whether BPD traits influence performance on specific elements of social cognitive tasks, i.e., positive/negative valence.

**Method:**

Community-based typical controls (*n* = 151; 51% female) were recruited through an online survey. Participants completed aspects of the Personality Assessment Inventory pertaining to BPD traits, the Interpersonal Reactivity Index, and measures of both emotion recognition and mentalising.

**Results:**

Following group stratification into high/low BPD traits, participants with high BPD traits were observed to perform significantly better when identifying negative valence stimuli. Furthermore, high levels of affect instability was found to significantly influence negative valence recognition.

**Conclusion:**

This research highlights previous research which shows a paradox between higher performance on measures of social cognition, with a group of individuals who report significant interpersonal and relational difficulties. This research supports the assessment of social cognitive processes for people with BPD and/or high BPD traits to support clinical formulation of strengths and difficulties.

## Introduction

Borderline personality disorder (BPD) is defined as a psychiatric condition, characterised by a pervasive pattern of marked impulsivity and instability in affects, self-image, and interpersonal relationships (1). BPD as a clinical syndrome affects up to 5.9% of the general population (2); 11% of psychiatric outpatients (3); and 33% of psychiatry inpatients (4), with an increasing incidence (5, 6).

The diagnostic criteria outlined by the DSM-5, requires an individual’s particular maladaptive personality traits to be pervasive, persistent, and unlikely to be limited to a particular developmental stage or another mental disorder (1). The DSM-5 also requires clinicians to consider two sets of criteria (A and B), in the assessment of BPD. Criterion A requires judgement of severity of identity problems, self-direction, empathy, and intimacy. Criterion B requires the presence of at least four out of seven pathological personality traits.

Borderline personality disorder traits include emotional lability, anxiousness, separation insecurity, depressivity, impulsivity, risk-taking, and hostility (1, 7). According to some, BPD has three clusters of symptoms which relate to the intra- and inter-personal nature of the disorder and encompass the range of diagnostic criteria of the DSM-5: affect dysregulation, behavioural dysregulation, and significant difficulties relating to others (8). Affect and behavioural dysregulation largely relate to the self, though significantly impact interpersonal relationships and are described as phenotypic traits of BPD in both cross-sectional and longitudinal studies (9, 10).

Clinical and empirical observations have proposed that one’s own impaired social cognition is a mechanism underlying the development and maintenance of BPD traits through suboptimal social encounters and engagements with others (11). Fonagy et al. (12) showed that impaired social relatedness and social cognition is linked to BPD traits. Social cognition is broadly defined as the ability to identify, understand, and interpret mental states and recognise emotions (13). Social cognition is a neurocognitive concept that includes comprehending others’ intentions, beliefs, feelings, and mental states (affective theory of mind), as well as social interaction, social context, and social decision making (cognitive theory of mind) (14). Empathy generally refers to a affective route for understanding others (15), while theory of mind (ToM) refers to neuropsychological processing of others’ mental states or intentions (16). The terms mentalising and ToM are often used interchangeably, with ToM typically recognised as a superordinate category, however it is important to note how these terms differ. Mentalising refers to elaboration of drive-affect experiences as mental phenomena and structures and is critical for comprehending each other and oneself in relation to subjective states and mental processes (17). ToM encompasses the ability of perspective taking to infer others’ thoughts, beliefs, and emotions as well as decoding others’ complex emotions and mental states by understanding subtle affective perceptual stimuli and contextual information (18, 19).

Social cognitive abilities are required for successful social interactions and enable individuals to develop and maintain both short and long-term relationships with others. According to Goueli et al. (20), impaired social cognition is a psychopathological cornerstone of BPD. Findings relating to mental state attribution are incongruous in the area of BPD. A myriad of studies assessed self-reported perspective taking in BPD patients using the Interpersonal Reliability Index [IRI; (21)], and found reduced performance when compared to people with anorexia nervosa and non-clinical controls (22–24). Emotion recognition tasks have also been used with inconsistent results (25, 26). Some report people with BPD correctly identified emotional facial expressions (25, 27), while others indicated that people with BPD had reduced performance and showed bias toward the perception of anger in pictures of faces displaying blends of two emotions (26). Bora et al. (18), suggest interpersonal problems and difficulties in processing social information in BPD can be best explained by patients’ maladaptive meta-social cognitive style and the top-down effects of such abnormalities as opposed to having a primary neuro-social cognitive deficit which may partially explain the variance in results in the literature. Notably, studies have indicated that when facial emotion recognition tasks approximate more complex and naturalistic situations, BPD patients display increased error rates compared to non-clinical controls (28, 29).

Ghiassi et al. (30) investigated mentalising, which was not impaired in BPD patients compared to non-clinical controls. However, other research reports deficits in ToM for people with BPD (23). This may be due to measurement and psychometric error, as the faux-pas task (31) was used by (23), while Ghiassi et al. (30) used the cartoon task (32) and the differences of measurement between social cognition tests is nuanced. Using the Movie for Assessment of Social Cognition [MASC; (33, 34)] BPD patients have been reported to have impaired recognition of emotion, thoughts, and intentions of others (35). The MASC was utilised in a further study on adolescents with BPD traits which found evidence for impaired social cognition in those with high traits compared to low (36). Notwithstanding the above, there is also a body of literature which suggests that people with BPD have a superior ability to infer mental states of others, when compared to typical controls. Such findings contribute to the self-other social cognitive paradox, which suggests that BPD patients have enhanced mentalising abilities, despite a fundamental difficulty with relatedness and interpersonal relationships (37). One such study (38) investigated outcomes on the “Reading the Mind in the Eyes Test” (RMET) in BPD patients compared to healthy controls. Currently, the wider literature is mixed regarding whether the RMET is a measure of emotion recognition, mentalising or a combination of both (39–41). In line with the view of Oakley et al. (40) who report that “theory of mind is not theory of emotion,” we consider emotion recognition tasks as those more “basic emotion” labelling or matching (e.g., happy/sad/angry), with mentalising considered to involve more complex, higher-level cognitive processing (42, 43).

This study reported the BPD group performed significantly better on total RMET score. A later study examining behavioural and neuropsychological responses of BPD patients and healthy controls during performance of the RMET supported these results (37). Results showed BPD patients demonstrated superior mental state discrimination than healthy controls. A significant main group effect was seen, specifically in mental state discrimination between positive and negative eye gazes. Unoka et al. (44) also utilised the RMET to research mentalising in BPD; This study used a sample of 78 BPD patients and 76 matched healthy controls and found poorer on the RMET when patients were compared to controls, though no significant difference was reported for negative items on the task. Petersen et al. (45) also report that people with BPD performed poorer on the RMET test, which was specifically driven by incorrect responses to positive stimuli. Zabihzadeh et al. (46) further investigated outcomes on the RMET and the Faux Pas Test (cognitive theory of mind) which showed that people with BPD had higher scores on mentalising, while the control group was higher on cognitive theory of mind. Savage and Lenzenweger (47) further studied participants with BPD traits comparing scores on RMET performance pre and post social exclusion *via* computerised task that mimics social ostracism. A significant interaction was found between participants with BPD traits and RMET scores, suggesting that once an individual with BPD traits experiences social exclusion, their objectivity decreases, and negative affective valence is ascribed to stimuli previously perceived as neutral. Similar results were observed by Scott et al. (48) who showed that patients with BPD tend to misattribute malevolence to benign social stimuli, including facial expressions, with enhanced accuracy on the RMET in healthy individuals with high BPD traits compared to low. These findings suggest BPD traits may be associated with enhanced ability to detect and interpret mental states and a bias for attributing negative emotions to non-negative stimuli. Notably, researchers have suggested the RMET to be a measure of superficial mentalising as opposed to a comprehensive measure of genuine mentalising ability due to the fact that there is no requirement for participants to reason about behaviour based on their mental state attributions (45). Such methodological flaws in measures of social cognitive abilities may partially account for the heterogenous findings in the area.

Incongruent results have also been reported for empathy, which can be described as an observer’s emotional response to another person’s emotional state (49). Harari and colleagues found that self-reported affective aspects of empathy were increased in BPD patients. Conversely, New et al. (24), did not find significant group differences when comparing BPD patients and non-clinical controls using the same measure. In further conflicting results, Dziobek et al. (50) found BPD patients had significantly reduced tendencies to feel empathy for others in emotionally distressing situations assessed using the Multifaceted Empathy Test [MET; (51)]. However, while there is no objective guideline as to what level of Cronbach’s alpha is required for an instrument to be considered useful, general conventions would characterise the reliability of the MET scale as inadequate (52, 53). Additionally, this study reported decreased values on the Interpersonal Reactivity Index [IRI; (21)], empathic concern scale.

It is clear from the literature that further research is needed into social cognition and both clinical and sub-clinical BPD traits. Previous studies on BPD have focussed narrowly on specific aspects of social cognition and have produced conflicting results (35, 54). The assessment of varied domains of objective and subjective social cognition is required to identify typical patterns of abilities as well as deficits. Research on social cognition has the potential to bolster psychopathology models of BPD that emphasise social cognitive outcomes as a core deficit (55). The current study aims to investigate the relationship between social cognition and BPD traits further, to determine which specific BPD traits predict social cognition outcomes. The objective was to determine if group differences existed on the specific positive, negative, and neutral subscales of the RMET, and whether performance related to other measures of social cognition. This study further aimed to quantify the predictive relationship between BPD traits and mentalising outcomes, in a community sample of controls.

## Materials and methods

### Participants and procedure

This study employed a cross-sectional survey-based design from a community-based sample of typical controls. Data from 151 participants were gathered using Prolific Academic^©^, an online platform for survey-based data collection. In terms of eligibility criteria, participants were required to be over the age of 18, to give explicit consent for data usage, and to be residents in the Republic of Ireland. Exclusion criteria included having existing neurological or mental health diagnoses which may interfere with test performance; and being non-native English speakers. Participants were screened for exclusion criteria through online survey questions, prior to engaging with the study. Following this, participants provided consent and demographic details and then proceeded to complete the online psychometric and cognitive measures.

On completion of the survey, participants received a gratuity of commensurate with the hourly minimum wage rate in Ireland. A pilot study was conducted with 10 participants, with no changes made following this. Consequently, the study was continued and the data from these 10 was retained. The average duration of the experiment was approximately 30 min. The mean age of participants was 38.79 (SD = 12.37), ranging from 20 to 76. The sample was comprised of 49.67% males (*N* = 75) and 50.33% females (*N* = 76).

This study was approved by the School of Psychology Health Research Ethics Committee at National University of Ireland Galway. All procedures were conducted in accordance with the principles expressed in the Declaration of Helsinki.

### Measures

#### Demographics

Participants provided basic demographic information such as age, sex, education, and employment status. Demographics, social cognition and psychopathy outcomes were gathered using the online platform, Prolific Academy^©^. This platform has been shown to have high data quality, a diverse participant pool, and demonstrates reproducibility of known effects.

#### The reading the mind in the eyes task

The Reading the Mind in the Eyes Task [RMET; (39)] is a 36-item assessment where black and white photographs of eye regions are presented, and participants are requested to infer mental states from four choices provided. The RMET can also provide individual scores for Positive, Negative, and Neutral valence (56–59). Examples of Positive valence include Friendly (Q20); Negative valence: Hostile (Q26); Neutral valence: pensive (Q24). The RMET has been found to be reliable and stable over time (60), with a Cronbach’s alpha of 0.88 (61). The RMET has further been validated using remote administration *via* survey-based platforms (62), and does not produce ceiling effects (63).

#### The Florida Affect Battery

The Florida Affect Battery [FAB; (64)], is a measure of emotion recognition. Five different emotional states are used across the subtests: happiness, sadness, anger, fear, and neutral. Subtests of the FAB included were (1) facial affect discrimination, and (2) facial affect naming. In the facial affect discrimination subtest participants must determine whether two faces depict the same or different emotional expressions. In the facial affect subtest, individual faces are shown as stimuli and participants is asked to name the emotion depicted. Test-retest reliability of the FAB has been examined and ranged from 0.89 to 0.97, with the Cronbach’s alpha for the facial scales reported at 0.82 (64).

#### The Interpersonal Reactivity Index (IRI)

The Interpersonal Reactivity Index [IRI; (21)] is a 28-item self-report instrument designed to assess empathic tendencies. The IRI consists of four separate 7-item subscales: Perspective Taking (PT; Cronbach’s alpha: 0.83), Fantasy (FS; Cronbach’s alpha: 0.86), Empathic Concern (EC; Cronbach’s alpha: 0.83), and Personal Distress (PD; Cronbach’s alpha: 0.78), which are measured using a 5-point Likert scale ranging from “Does not describe me well” to “Describes me very well” (65). PT refers to the tendency to spontaneously adopt the psychological point of view of others. FS describes the likelihood that a person identifies with a fictional character. EC assesses individuals’ feelings of concern and compassion for others. Lastly, PD indicates the extent that a person feels uneasiness or worry when exposed to the negative experience of others. The IRI has robust validity and is among the most widely used measures of empathy (66).

#### The Personality Assessment Inventory

The Personality Assessment Inventory [PAI; (67, 68)], is a self-administered test of personality and psychopathology. The PAI is a 344-item questionnaire in which there are 22 non-overlapping subscales. For the purpose of this study, Borderline Features (Bor) was measured, which focuses on attributes indicative of a BPD, including unstable and fluctuating interpersonal relations, impulsivity, affective lability and instability, and uncontrolled anger. The Bor scale is a sum of four subscales: Bor-A (Affect Instability); Bor-I (Identity problems); Bor-N (Negative Relationships), and Bor-S (Self-Harm). The respondent is asked to check one of four response options indicating the extent to which the item statement accurately describes them. For each scale responses are standardised with reference to a national census-matched sample of community adults. The standardisation results in a T score, with 50T representing the mean, and the standard deviation being 10T. A score of ≥ 70T represents a level of reported symptoms that is rarely seen in the general population and is considered very clinically relevant. For the purpose of this study, people who scored > 70T on a measure of the PAI Borderline subscales, were categorised as high self-report, compared to those who endorsed items < 70T. The PAI was chosen for this study as it has robust content and discriminant validity as well as internal consistency reliability estimates (69, 70), with the Cronbach’s alpha of the Bor scale at 0.91 (71).

### Statistical analysis

Demographic characteristics and outcome data are reported as means, standard deviations, and frequencies as relevant. Based on the data obtained, classification for good internal consistency, using Cronbach’s alpha, remains at the internationally accepted value > 0.70. The data was analysed using the IBM SPSS v27. Power analysis revealed 150 participants were required to detect a minimum effect size (*r* = 0.15) with an alpha level of 0.05 with 95% power. Our *a priori* power analyses for group comparisons indicated that a minimum of = 42 would be required per group to detect a medium effect size (power = 0.8; *f* = 0.25, α = 0.05, λ = 8.0). Descriptive statistics, bivariate correlations, multivariate ANOVA, general linear regression, and hierarchical multiple regression were conducted to analyse the data. As above, PAI Borderline subscales were used to determine if an individual scored high (> 70T) or low (< 70T). The significant predictor variables were regressed onto RMET using hierarchical multiple regression.

## Results

### Correlations

Pearson’s product moment correlation coefficient was conducted to investigate the relationship between the BPD traits and social cognitive outcomes, as shown in Table 1. There was a significant relationship between the PAI Borderline total, with the personal distress subscale of the IRI, (*r* = 0.33, *p* < 0.001). Bor-A was significantly related to the RMET total score, (*r* = 0.17, *p* < 0.001), more specifically, the percentage correct on the RMET negative valence, (*r* = 0.28, *p* < 0.001). Bor-A also correlated negatively with IRI perspective taking (*r* = -0.16, *p* < 0.05) and IRI personal distress (*r* = 0.37, *p* < 0.001). Borderline Identity Problems (Bor-I) positively correlated with IRI personal distress (*r* = 0.28, *p* < 0.001). A further significant relationship was found between Bor-N and IRI personal distress (*r* = 0.25, *p* < 0.001). There were no significant correlations between Bor-S and social cognitive outcomes.

**TABLE 1:** Pearson’s correlation statistics for BPD trait and social cognition variables.

Variables	1	2	3	4	5	6	7	8	9	10	11	12	13	14	15
1. PAI Bord. total	–														
2. BOR A total	0.75**	–													
3. BOR I total	0.82**	0.54**	–												
4. BOR N total	0.82**	0.53**	0.57**	–											
5. BOR S total	0.60**	0.27**	0.30**	0.23**	–										
6. IRI total	0.11	0.12	0.14	0.10	–0.02	–									
7. FAB affect total	–0.12	0.03	–0.14	–0.16	–0.05	0.05	–								
8. FAB discrimination total	–0.12	–0.14	–0.08	–0.07	–0.10	–0.03	0.20*	–							
9. RMET total	–0.02	0.17*	–0.01	–0.03	–0.16	0.08	0.23**	0.09	–						
10. RMET valence positive	–0.10	0.02	–0.08	–0.04	0.03	0.03	0.26**	0.22**	0.78**	–					
11. RMET valence neutral	–0.10	0.10	–0.08	–0.14	–0.14	0.05	0.15	–0.02	0.72**	0.39**	–				
12. RMET valence negative	0.14	0.28**	0.13	0.07	–0.03	0.10	0.08	–0.05	0.71**	0.22**	0.35**	–			
13. IRI fantasy	0.06	0.10	0.09	0.02	–0.03	0.71**	0.15	–0.10	0.13	0.09	0.09	0.11	−		
14. IRI empathic concern	–0.01	–0.01	0.02	–0.02	–0.03	0.73**	–0.01	0.05	0.13	0.10	0.10	0.09	0.36**	–	
15. IRI perspective taking	–0.11	−0.16*	–0.04	–0.06	–0.08	0.67**	0.02	0.01	0.06	0.08	0.01	0.03	0.31**	0.51**	–
16. IRI personal distress	0.33**	0.37**	0.28**	0.25**	0.10	0.38**	–0.05	–0.04	–0.10	−0.170*	–0.04	0.02	0.07	–0.01	–0.14

**p < 0.01 level (two-tailed); *p < 0.05; N = 151; PAI, Personality Assessment Inventory; BOR, Borderline – A (Affect Instability), I (Identity Problems), N (Negative Relationships), S (Self-harm); FAB, Florida Affect Battery; IRI, Interpersonal Reactivity Index; RMET, Reading the Mind in the Eyes Test.

### Analysis of variance

A one-way multivariate analysis of variance (MANOVA) was conducted to investigate performance in the RMET, grouped by high (> 70T; *n* = 53) or low (*n* = 98) reported scores. The dependent variables were percentage correct on RMET positive, negative, and neutral valence. Of each of the subscales, the MANOVA found significant differences between groups when stratified by Bor-A [Wilk’s Λ = 0.95, *F*_(3_,_147)_ = 2.88, *p* < 0.001, η^2^_*P*_ = 0.55] only. Subsequently, one-way ANOVAs for each valence subscale were conducted grouped by high/low Bor-A outcomes. Bonferroni correction was applied to control for multiple comparisons with an adjusted alpha level (α = 0.017). A significant difference between groups in the percentage correct on RMET negative valence was observed [*F*_(1_,_149)_ = 6.31, *p* < 0.017, η^2^_*P*_ = 0.04]. No significant differences were found between groups on the RMET positive valence [*F*_(1_,_149)_ = 0.82, *p* = 0.368, η^2^_*P*_ = 0.005], or neutral valence [*F*_(1_,_149)_ = 0.033, *p* = 0.856, η^2^_*P*_ = 0.001] after correcting for multiple comparisons. There was also a significant difference in the self-reported personal distress subscale of the interpersonal reactivity index, when stratified by high/low Bor-A [*F*_(1_,_149)_ = 19.24, *p* < 0.0001], with means and standard deviations presented above in Table 2.

**TABLE 2:** Means (M) and Standard Deviations (SD) on outcome measures for the total group, and stratified by Bor-A outcomes.

	Borderline – Affect Instability					
Outcome measures	Below 70T (n = 98)		Above 70T (n = 53)		Total group (N = 151)	
	*M*	*SD*	*M*	*SD*	*M*	*SD*
Age	39.76	12.64	37.02	11.76	38.79	12.36
FAB discrimination	4.24	0.80	4.00	0.91	4.15	0.849
FAB affect correct	17.72	1.72	17.56	2.08	17.66	1.85
RMET total	25.83	3.79	26.33	4.52	26.01	4.05
RMET Positive valence % correct	75.13	12.63	73	15.89	74.38	13.83
RMET negative valence % correct	66.41	14.92	72.57	13.34	68.57	14.65
RMET neutral valence % correct	74.05	19.61	74.66	19.37	74.27	19.66
IRI total	95.5	11.35	98.69	10.71	96.62	11.20
IRI fantasy	23.05	4.64	24.28	4.46	23.48	4.60
IRI empathic concern	27.80	4.11	27.56	4.29	27.71	4.16
IRI perspective taking	26.00	4.69	24.84	4.63	25.60	4.69
IRI personal distress	18.54	11.35	21.88	4.58	19.71	4.56
Bor-A*	6.02	1.55	10.75	1.68	7.68	2.77
Bor-I	5.40	3.28	9.11	3.76	6.70	3.87
Bor-N	5.56	3.37	9.32	3.49	6.88	3.85
Bor-S	3.12	2.88	4.35	3.55	3.55	3.18

*The Bor-A variable is the grouping variable for the sub-stratification. BOR; Borderline – A (Affect Instability), I (Identity Problems), N (Negative Relationships), S (Self-harm); FAB, Florida Affect Battery; IRI, Interpersonal Reactivity Index; RMET, Reading the Mind in the Eyes Test. Bold indicated significant between group differences.

### Regression

To investigate this further, a series of general linear regressions were conducted to determine the variance in valence recognition. The Bor-A subscale significantly predicted variance in the RMET negative valence [*F*_(1_,_149)_ = 12.3, *p* < 0.001, *R*^2^ = 0.08, *R*^2^_*adjusted*_ = 0.07], but not positive or neutral. A hierarchical multiple regression was conducted to examine if scores on each PAI Borderline subscales (Bor-A, Bor-I, Bor-N, and Bor-S) further predict the percentage of correct answers on the RMET negative valence measure, while controlling for age and sex. The predictor variables of age and sex were entered into the first block and the PAI Borderline subscale scores were entered into the second block. The criterion variable was percentage of correct answers on the RMET negative valence measure.

Multicollinearity was not present in the data as observable in Table 1. The variance inflation factor scores were less than 10 (range = 1–1.73) and tolerance scores were greater than 0.1 (range = 0.58–1). The results of the hierarchical multiple regression, as shown in Table 3 below, show that the overall model was significant, accounting for 5.7% of variance in RMET negative valence percentage correct [*F*_(6_,_144)_ = 2.50, *p* < 0.05, *R*^2^ = 0.09, adjusted *R*^2^ = 0.06]. Step one age and sex, did not contribute significantly to the model [*F*_(2_,_148)_ = 0.17, *p* = 0.85, Δ*R*^2^ = 0.02, adjusted Δ*R*^2^ = 0.01]. Step two, Bor-I, Bor-N, Bor-S did not significantly contribute to the model [*F*_(3_,_145)_ = 1.05, *p* = 0.37, Δ*R*^2^ = 0.02, adjusted Δ*R*^2^ = -0.01]. Step three, with the inclusion of Bor-A, significantly contributed to the model, explaining 9.4% of variance in RMET negative valence percentage correct [*F*_(1_,_144)_ = 11.3, *p* < 0.001, Δ*R*^2^ = 0.07, adjusted Δ*R*^2^ = 0.05]. Bor-A was the only significant contributor to the variance explained (β = 0.34, *p* < 0.001).

**TABLE 3:** Summary of hierarchical regression model.

Step	Variable	β	Δ *R*^2^	Adjusted Δ *R*^2^	*F* change
1	Sex	0.04			
	Age	–0.03	0.02	0.01	0.17
2	Bor-I	0.04			1.05
	Bor-N	–0.11			
	Bor-S	–0.10			
3	Bor-A	0.34**	0.07	0.05	11.3**

Total *R*^2^ = 0.09, adjusted *R*^2^ = 0.06; **p < 001; BOR; Borderline – A (Affect Instability), I (Identity Problems), N (Negative Relationships), S (Self-harm).

## Discussion

This study investigated the relationship between BPD traits and social cognitive outcomes in a non-clinical community-based sample (*n* = 151). BPD traits were measured using the Borderline scale of the PAI, a clinically validated and reliable measure. Social cognitive abilities were evaluated using three measures, each assessing a different aspect of social cognition: the RMET (mentalising/facial emotion recognition), the IRI (empathy), and the FAB (emotion recognition). It was hypothesised, in line with previous literature, that higher BPD trait scores would significantly relate to social cognitive abilities. A further aim was to investigate what traits effect what social cognitive skills in addition to examining the predictive value of this relationship.

The above results indicate that a statistically significant relationship is present between elevated specific BPD traits (affect instability) and social cognitive outcomes (mentalising). These findings are highly consistent with previous research showing elevated BPD traits and improved aspects of social cognition (38, 44, 46–48, 72). While not significantly different relative to typical controls, the current study findings pertaining to valence recognition support similar findings of Anupama et al. (73), who found facial emotion recognition ability was significantly lower for patients diagnosed with BPD for the eye region associated with positive and neutral valences. The results of the present study are congruous with those reported by Arntz et al. (74), and Fertuck et al. (38), which show improved performance on mentalising tasks for people with BPD. This enhanced ability, or tendency to over attribute extreme mental states to others may be referred to as over-mentalising or hyper-mentalising, as reported by Ortega-Díaz et al. (75) and Sharp et al. (36). Sharp and colleagues and Sharp and Vanwoerden (76); Sharp and Vanwoerden (77) suggest that borderline features do not associate with deficits in mentalising, but rather an altered style of mentalising in the form of hyper-mentalising. The current findings provide support for aspects of hyper-mentalising to be considered as a core feature of BPD.

Numerous studies have examined clinical cohorts with BPD utilising the IRI. Guttman and Laporte (22), found impairments in perspective taking in BPD patients when compared to patients with anorexia nervosa and non-clinical controls. Additionally, Dziobek et al. (50), reported BPD patients to have significantly reduced tendencies to feel empathy for others in distressing situations. While there was no significant difference on these subscales for our non-clinical sample, there was a significant negative association between high affect instability and the IRI perspective taking subscale, despite Bor-A being associated with better performance on mentalising. This self-other paradox may suggest that some people with high borderline traits have intact, or even enhanced ability to infer mental states and intentions of others, though may have an incongruent self-impression of their social cognitive abilities. This may relate to previous experience and learned behaviour following social interactions. These results could support development of interventions to address such a disparity between one’s self-impression of and actual abilities. Furthermore, BPD patients can display a bias toward the perception of anger in pictures of faces displaying blends of two basic emotions (26). As BPD and BPD traits are associated with higher levels of adverse experiences, it may also be possible that individuals with high BPD traits have higher exposure to negative valence in a social or environmental context, and as such can identify and recognise it more accurately than typical controls, or those with low BPD traits. This may relate to hypervigilance, whereby individuals with higher BPD traits may have a propensity to analyse the environment for threat in the form of hostile behaviour, i.e., negative valence. This would be an avenue for future research to explore in detail. Importantly, this study showed the need for more detailed social cognitive testing for people presenting to mental health services, as well as people with psychiatric presentations. This may tell us more about social cognition from a theory perspective, as well as inform interventions such as social or cognitive remediation which may be useful from a clinical perspective.

The results of this study are congruent with the literature on BPD that assigns a high degree of importance to affect instability. Previous research has suggested that affect instability is the core pathology in BPD (78). Results highlight that participants’ affect instability score accounted for 7% of variance in their percentage correct on negative valence. Furthermore, the overall model was found to predict 5.7% of variance, with the affect instability variable being the sole significant contributor.

Notable strengths of the current study are the broad age range and the gender balance of the sample which contribute to the generalisability of the results. Additionally, the use of a range of measures including the RMET valence subscales contribute to the relevance and novelty of the research. While this study has yielded numerous significant results, these findings are subject to certain limitations. Firstly, the self-report nature of this study must be considered; The use of self-report has limitations such as social desirability bias, misinterpretation of the questions, and the restrictive nature of some scales. A second limitation is the use of a non-clinical cohort to measure BPD traits, over and above clinical symptoms and cognitive outcomes in people with a clinical diagnosis.

Further assessments are required to contextualise the findings of this research and begin to offer more causative reasons for these findings, with larger samples, longitudinally. Firstly, investigating the relationship between BPD traits and predisposing factors may yield further information as to why there are elevated BPD traits, as well as offer a contextual explanation for the findings in this study. Researchers utilising the scales employed in this study should note the lack of significant results when analysing the *total* scores of the measures, i.e., RMET and IRI, and consider more specific subscales. Analysing the subscale totals provides a more detailed and comprehensive understanding of the relationship being studied and yields significant results where the overall total does not. This is pertinent when investigating a disorder such as BPD as symptomology and presentation can vary greatly between patients. Lastly, in relation to the psychometric measurement of cognitive domains, as noted above, there is ongoing debate within the literature as to whether the RMET is a measure of emotion recognition, theory of mind, or whether it combines features of both (39–41). Based on the pattern of outcomes within this study, there is evidence to suggest that the RMET measures something additional, if not arguably entirely different, to emotion recognition alone. Future research could consider this convergence and divergence further through prospective item-level analyses of the RMET alongside measures of emotion recognition. Future studies may consider utilising additional or alternative measures of social cognition to bolster ecological validity as it has been proposed that the RMET is a measure of superficial mentalising (45). The MASC has been reported to be a more complex and ecologically valid measure which may better identify impairments in social cognition (34).

The data indicates there is a significant relationship between social cognitive function and BPD traits. Different traits were found to correlate higher than others with certain social cognitive skills. Specifically, this study suggested high affect instability predicts recognition of negative valence. Due to the cross-sectional survey-based nature of this study, a causal hypothesis between social cognitive performance and elevated borderline personality disorder traits cannot be examined, however, this may be an avenue for future research.

## Data availability statement

The raw data supporting the conclusions of this article will be made available by the authors, without undue reservation.

## Ethics statement

The studies involving human participants were reviewed and approved by the School of Psychology, National University of Ireland Galway. The patients/participants provided their written informed consent to participate in this study.

## Author contributions

MKG, GC, and TB analysed the data. All authors contributed to the development of the study, data collection, and manuscript development and review.
